# Retinal Defocus and Form-Deprivation Exposure Duration Affects RPE BMP Gene Expression

**DOI:** 10.1038/s41598-019-43574-z

**Published:** 2019-05-14

**Authors:** Yan Zhang, Eileen Phan, Christine F. Wildsoet

**Affiliations:** School of Optometry, University of California, Berkeley, Berkeley, CA USA

**Keywords:** Gene expression, Genetics research

## Abstract

In the context of ocular development and eye growth regulation, retinal defocus and/or image contrast appear key variables although the nature of the signal(s) relayed from the retina to the sclera remains poorly understood. Nonetheless, under optimal visual conditions, eye length is brought into alignment with its optical power to achieve approximate emmetropia, through appropriate adjustment to eye growth. The retinal pigment epithelium (RPE), which lies between the retina and choroid/sclera, appears to play a crucial role in this process. In the investigations reported here, we used a chick model system to assess the threshold duration of exposure to lens-imposed defocus and form-deprivation necessary for conversion of evoked retinal signals into changes in BMP gene expression in the RPE. Our study provides evidence for the following: 1) close-loop, optical defocus-guided (negative and positive lenses) bidirectional BMP gene expression regulation, 2) open-loop, form-deprivation (diffusers)-induced down-regulation of BMP gene expression, and 3) early, transient up-regulation of BMP gene expression in response to both types of lens and diffuser applications. The critical exposure for accurately encoding retinal images as biological signals at the level of the RPE is in the order of minutes to hours, depending on the nature of the visual manipulations.

## Introduction

Many animals, including humans, are born with refractive errors, which represent mismatches between the axial length and optical power of the eye^1,2^. Typically, these early mismatches are subsequently corrected and the status maintained through a developmental process known as emmetropization, involving coordinated growth of the eye and its optical power^2,3^. Failure of this emmetropization process results in two common types of refractive errors - myopia (near-sightedness), where the eye is too long for its optical power and hyperopia (far-sightedness), where the eye is too short for its power.

**Figure 1:**
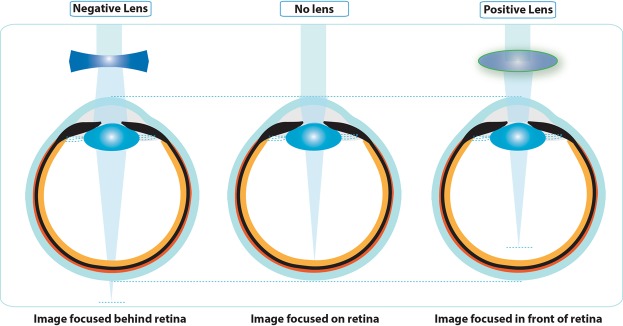
Schematic diagram illustrating the effects of imposed optical defocus on the location of ocular images of distant objects for an emmetropic eye (center); negative lenses move the image plane behind the retina, imposing hyperopic defocus (left) while positive lenses move the image plane in front the retina, imposing myopic defocus (right).

While dysregulated eye growth may be the result of genetically programmed altered growth, there is also strong evidence for visual environmental influences and gene-environment interactions^2–4^. Convincing evidence for visual environmental influences on postnatal eye growth regulation is provided by experiments encompassing a range of animal models, including chick, guinea pig, tree shrew, and monkey, and three types of visual manipulations, form-deprivation, in which retinal image contrast is attenuated across some or all spatial frequencies, and optical defocus, in which lenses are used to shift the optical plane of focus for distant objects relative to the retina, either behind it (negative lenses) or in front of it (positive lenses) (Fig. 1)^2^. Both form-deprivation and negative lenses (imposed hyperopic defocus) accelerate axial elongation while positive lenses (imposed myopic defocus) slow eye growth. Regulation appears largely local to the eye, involving multiple ocular tissues, including but not limited to the retina and retinal pigment epithelium (RPE). While growth changes have also been well characterized at the organ (eye size) and tissue levels (choroidal thickness changes, scleral remodeling), nonetheless the details of the assumed retina-sclera signaling cascades are poorly understood at the molecular level, with many questions remaining unresolved^2,3,5–8^. Two key unresolved questions are: (1) how does the retina decode changes in retinal images effected by visual manipulations, and (2) how are derived retinal signals relayed across the RPE to effect changes in the choroid and sclera, which together determine the position of the retina and thus the state of focus (refractive error) of the eye^2,3,6^.

Here, we studied gene expression changes in the RPE, which, being strategically positioned between the retina and choroid/sclera, is a necessary component of any growth-regulating retina-sclera signaling cascade. Studies previously identified, using young chicks as a model of ocular growth regulation, three BMPs - BMP2, 4, and 7, all of which showed consistent gene expression changes in RPE in the presence of retinal defocus, with bidirectionality profiles consistent with the direction of altered eye growth^9–11^. The observation of down-regulation of BMP2 gene expression with imposed hyperopic defocus parallels trends in combined retina/RPE samples described in an earlier study involving form-deprived chicks^12^. The current study included form-deprivation, −10 D lenses to impose hyperopic defocus, as well as three positive lens powers (+10, +20 & +30 D), to impose varying levels of myopic defocus, with treatment duration included as an additional variable in all cases. The results as reported here for our profiling study of the temporal stimulus requirements for related BMP gene expression changes in chick RPE, confirm early findings in relation to the encoding of defocus, i.e., that BMP gene expression encodes the sign of imposed optical defocus, with positive and negative lenses inducing up- and down-regulation respectively. New insights include: (1) BMP gene expression is down-regulated by form-deprivation (diffusers), as with negative lenses, (2) BMP gene expression shows early, transient up-regulation in response to all treatments, both positive and negative lenses, and diffusers, and (3) there are critical visual stimulus-dependent differences in minimum exposure durations required for generating consistent biological signals at the level of the RPE gene expression, ranging from minutes to hours.

## Results

### BMP gene expression in normal chick RPE

**Figure 2:**
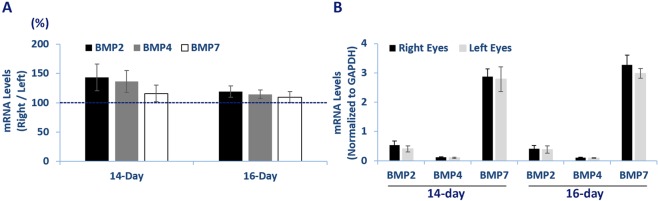
BMP2, BMP4, BMP7 gene expression in RPE from 14- and 16-day old chicks. There is no difference between right vs. left eyes or age-related difference in gene expression.

For the three genes of interest, BMP2, BMP4, and BMP7, baseline expression levels were measured in RPE from untreated chicks that matched in age, those subjected to monocular visual manipulations. All three genes showed detectible expression relative to GAPDH, with BMP7 showing the highest expression. As expected, expression levels were similar for right and left eyes, for both 14- and 16-day old groups (n = 8 & 7 resp. *p* > 0.05 in all cases; Fig. 2); there was also no age-related difference in expression (*p* > 0.05).

### Optical defocus-induced, sign-dependent bidirectional BMP gene expression in RPE

**Figure 3:**
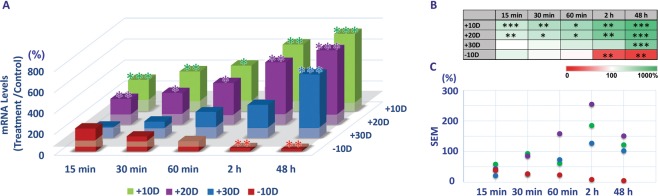
Differential gene expression of BMP2 in RPE with optical defocus treatments (+10, +20, +30 & −10 D lenses worn for 15 min to 48 h) (**A**). All three positive lens treatments induced up-regulation of BMP2 gene expression, while expression was down-regulated with the negative lens treatment, albeit in all cases varying in magnitude with lens power and/or exposure duration. (**B**) Heatmap summarizing differential gene expression changes with relevant statistics. (**C**) Variability expressed as SEMs, in gene expression changes shown in A; changes induced by positive lenses show greater variability than those induced by negative lenses. *p < 0.05; **p < 0.01; ***p < 0.001.

**Figure 4:**
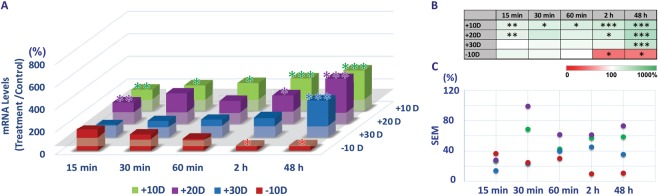
Differential gene expression of BMP4 in RPE with optical defocus treatments (+10, +20, +30 & −10 D lenses, worn for 15 min to 48 h). (**A**) All three positive lens treatments induced up-regulation of BMP4 gene expression, while expression was down-regulated with negative lens treatments, albeit in all cases varying in magnitude with lens power and exposure duration. (**B**) Heatmap of differential gene expression changes with relevant statistics. (**C**) Variability expressed as SEMs, in gene expression changes shown in A; changes induced by positive lenses show greater variability than those induced by negative lenses. *p < 0.05; **p < 0.01; ***p < 0.001.

**Figure 5:**
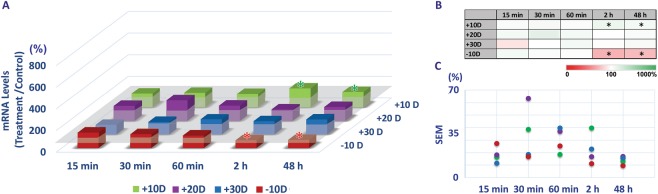
Differential gene expression of BMP7 in RPE with optical defocus treatments (+10, +20, +30 D & −10 D lenses worn for 15 min to 48 h). (**A**) +10 D lens treatments induced up-regulation of BMP7 gene expression, while expression was down-regulated with −10 D lens treatments. (**B**) Heatmap of differential gene expression changes with relevant statistics. (**C**) Variability expressed as SEMs, in gene expression changes shown in A; changes induced by positive lenses show greater variability than those induced by negative lenses. *p < 0.05.

The critical (threshold) treatment period required for consistent optical defocus-induced, sign-dependent BMP gene expression changes in 14 day-old chicks varied according to both the lens power and sign (positive or negative). Of the three different powers of positive lenses (+10, +20, +30 D), all induced rapid up-regulation of BMP2, BMP4, and BMP7 gene expression in the RPE relative to expression in fellow untreated eyes, with changes detectible after as little as 15 minutes of treatment. In contrast, down-regulation in the expression of the same three BMP genes, as characteristic of responses to negative lenses, required 2 h of exposure in the case of the −10 D lens tested (Figs 3–5, Supplemental Table 1).

For BMP2, the +10 and +20 D lenses induced similar patterns of gene expression changes (Fig. 3A); up-regulation was detectable after as little as 15 min, albeit increasing with exposure time to reach 739 ± 121% (*p* < 0.001, n = 8) and 703 ± 151% (*p* < 0.001, n = 11) respectively with 48 h of exposure. With a further increase in positive power, i.e., with +30 D lenses, 48 h of exposure was required for detectible up-regulation of BMP2 gene expression (582 ± 102%, *p* < 0.001, n = 11) (Fig. 3A). For −10 D lenses, a minimum of 2 h of exposure was required to induce significant down-regulation of BMP2 gene expression, with 48 h of exposure having a similar effect (31 ± 8%, *p* < 0.01, n = 9, 2 h; 23 ± 5%, *p* < 0.01, n = 8, 48 h) (Fig. 3A). A heatmap summarizing the effects of lens power, sign, and treatment duration on BMP2 gene expression is shown in Fig. 3B.

All actively regulated, homeostatic mechanisms are expected to have defined operating ranges. For the emmetropization mechanism targeted here, the range of imposed defocus stimuli that can be accurately decoded presumably reflects that encountered during normal development. In the context of ocular growth regulation, it is also likely that, there is a threshold sampling time required for encoding of defocus that increases with the magnitude of defocus, to minimize the effects of defocus experienced simply as consequence of objects being near or far relative to the point of regard. In the current study, chicks were not restrained, and their visual world, thus neither restricted nor static. Thus, for the highest positive power (+30 D) lens, which has a focal length of 3.33 cm, the experience of relatively in-focus and slightly blurred images would have been limited in objects in close proximity and thus also very limited in time. For the −10 D lens, only active (lenticular) accommodation could have reduced the experience of blur^13^. It is possible that for higher levels of defocus the direction of response is random, i.e., trial and error, in which case we would expect variable gene expression changes, at least initially. Or perhaps there is a default direction to the elicited response. When the lenses are left in place for a sufficiently long period of time, compensatory changes in the choroid and sclera will attenuate the imposed defocus, more quickly with low powered lenses, after which gene expression would also be expected to normalize. As one approach to test these ideas, we analyzed the variability in the results for BMP2 from the current study. Thus for the reported gene expression data (ratio of gene expression in treated versus fellow control eyes, expressed as percentage), the Standard Error of Mean (SEMs) were calculated and plotted (Fig. 3C). These data are also summarized in Supplemental Table 1. Interestingly, with the −10 D lens treatment, the variability was relatively consistent across all exposure durations. In contrast, with the positive lenses, the variability in gene expression changes were greater with 30 min of exposure than with 15 min of exposure, even though the sign of defocus appeared to have been accurately encoded, i.e., up-regulation of RPE gene expression was observed, even with the shortest (15 min) exposure duration. The larger variability in BMP2 gene expression levels with positive lenses suggests greater variability in the magnitude and/or sign of retina-derived signals reaching the RPE, and may reflect the inherent variability in the myopic defocus experience when chicks are not restrained.

BMP4 and BMP7 genes showed similar trends with respect to expression changes to those described above for BMP2, albeit smaller in magnitude in both cases (Figs 4 and 5).

### Form-deprivation-induced down-regulation of BMP gene expression in RPE

**Figure 6:**
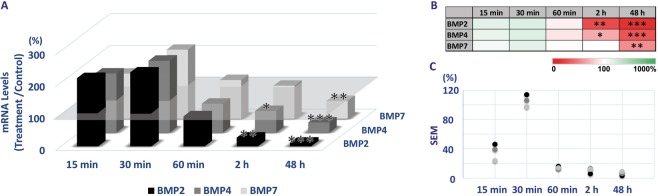
Differential gene expression of BMP2, 4, and 7 in RPE with monocular form-deprivation maintained for 15 min to 48 h. (**A**) Significant down-regulation in expression was detected with 2 and 48 h of treatment for BMP2 and BMP4 genes and after 48 h of treatment for BMP7. Heatmap of differential gene expression changes with relevant statistics. (**B**) Variability expressed as SEMs, in gene expression changes shown in A; it tends to be smaller with longer (60 min–2 h) exposures. (**C**) *p < 0.05; **p < 0.01; ***p < 0.001.

With the monocular diffuser treatment, both BMP2 and BMP4 genes showed down-regulation in their expression with 2 and 48 h exposures (Fig. 6, Supplemental Table 1). Down-regulation of BMP7 gene expression was also observed but as with both lens treatments, the changes were much smaller than those recorded for both BMP2 and BMP4, and only reached significance after 48 h of exposure to the diffuser treatment.

BMP2 gene expression was significantly down-regulated in form-deprived eyes, after both 2 and 48 h of treatment, to 27 ± 6 and 12 ± 3%, of the expression in contralateral control eyes respectively (*p* < 0.01, n = 9; *p* < 0.001, n = 8), while shorter exposures were without effect. For the form-deprivation-induced changes in BMP2 expression, the greatest variability (SEM) is seen with the short, 30 min exposure, when the changes also did not reach statistical significance. The variability was much smaller for longer (60 min–48 h) exposures (Fig. 6C; Supplemental Table 1). Similar temporal gene expression profiles were recorded for BMP4 and BMP7 (Fig. 6C, Supplemental Table 1).

### Up-regulation of BMP gene expression in RPE in response to brief visual (optical defocus or form-deprivation) manipulations

Interestingly, when visual manipulations were limited to very short exposures, up-regulation of BMP2 gene expression was consistently observed, irrespective of the nature of the treatment, i.e., positive or negative lenses or diffusers (Table 1). Similar trends were also observed for BMP4, except for the −10 D lens treatment, which was without effect. Notably, BMP7 gene expression was relatively insensitive to very short visual manipulations, with the one exception being of the +30 D lens treatment, which induced up-regulation after just 5 min of exposure. These gene-dependent differences in responses to brief visual manipulations are consistent with the generally more robust responses, i.e., changes in gene expression, recorded in other experiments for BMP2 compared to BMP4 and to BMP7, which consistently showed the least robust response.

**Table 1:** Effects of very short (5 or 15 min) visual (optical defocus & FD) manipulations on BMP gene expression; mean ratios of levels in treated eye/contralateral eye expressed as percentage (%, SEMs in brackets).

	BMP2		BMP4		BMP7	
	Duration (min)	Change (%)	Duration (min)	Change (%)	Duration (min)	Change (%)
+10 D (n = 11)	15	295.4 [58.5]***	15	188.0 [27.5]**	5 or 15	NS
+20 D (n = 9)	15	241.6 [43.3]**	15	188.1 [28.1]**	5 or 15	NS
+30 D (n = 10)	5	291.4 [52.9]**	5	241.7 [42.7]***	5 min	162.8 [23.3]**
−10 D (n = 14)	15	206.4 [37.8]**	5 or 15	NS	5 or 15	NS
FD (n = 11)	15	213.8 [46.1]*	15	184.7 [38.9]*	5 or 15	NS

FD, form-deprivation; NS, no statistical significance; *p < 0.05, **p < 0.01, ***p < 0.001.

### Parallels between effects on BMP gene expression in RPE of imposed hyperopic defocus and form-deprivation treatments

**Figure 7:**
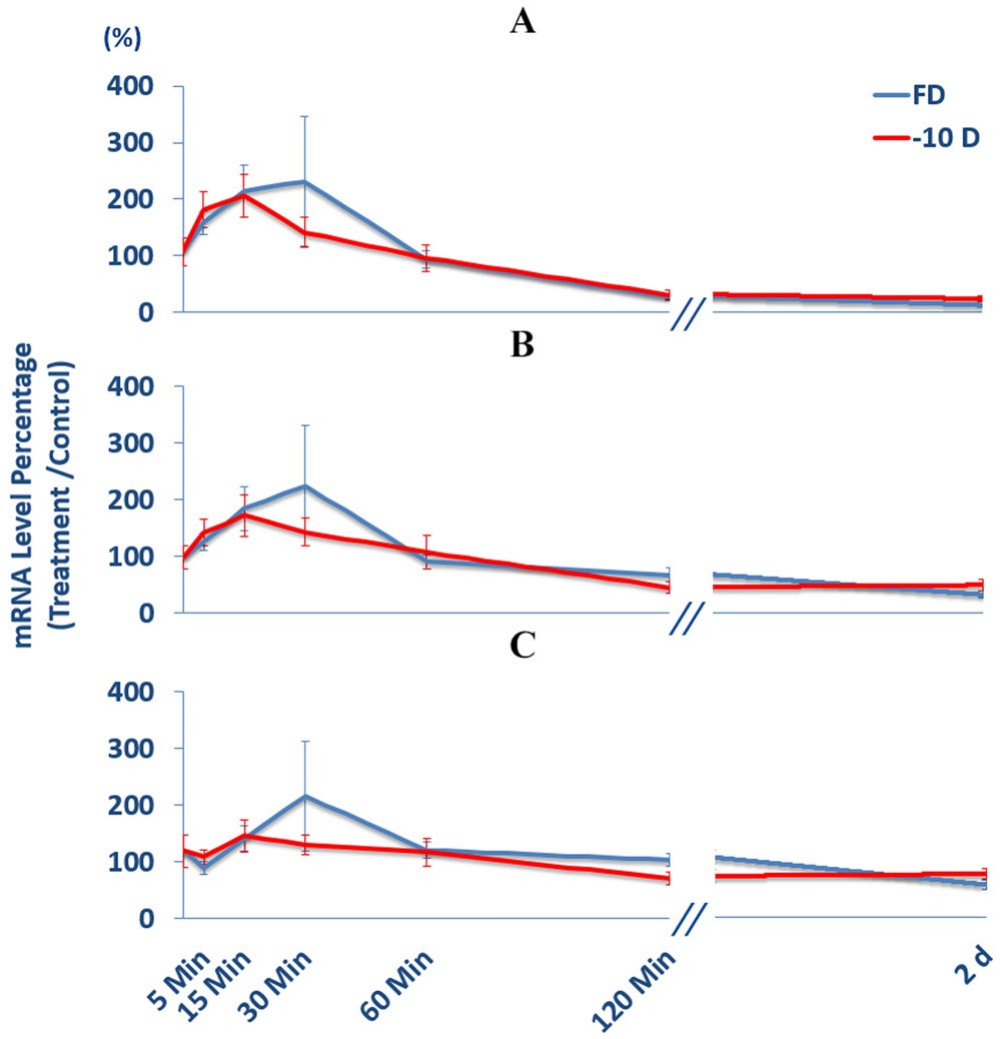
Gene expression changes in BMP2 (**A**), BMP4 (**B**), BMP7 (**C**) induced by −10 D lens and form-deprivation (FD) treatments. No statistically significant treatment-related differences were detected for any of the exposure durations tested (5, 15, 30, 60 min, 2 & 48 h). The large variability in the 30 min FD data originates from 2 of 12 birds, which showed increases of 1358 & 601% compared to the group mean of 232% for BMP2.

As negative lenses (imposed hyperopic defocus) and form-deprivation both reliably induce myopia under experimental conditions in young chicks, it is of interest to compare related induced BMP gene expression patterns, by way of understanding how interchangeable they are. For all three BMPs studied, i.e., BMP2, BMP4, and BMP7, both −10 D lens and diffuser treatments induced similar gene expression patterns, as illustrated graphically in Fig. 7. Furthermore, the gene expression changes induced by these two visual manipulations proved to be not significantly different, for any of the exposure times tested (5, 15, 30, 60 min, 2 & 48 h; *p* > 0.05 in all cases).

## Discussion

Using young chicks as an animal model of ocular growth regulation combined with visual manipulations (optical defocus and form-deprivation) to perturb normal emmetropization, we obtained further supporting evidence for roles of bone morphogenetic proteins (BMPs) and the RPE, as part of a presumed retina-sclera growth modulating signaling cascade. The data presented here also provide new insight into the critical exposure periods required for encoding these altered visual experiences via changes in BMP gene expression in the RPE. The main contributions of this study can be summarized as follows: First, we were able to confirm that for BMP2, 4, and 7, RPE gene expression is differentially regulated by three types of visual manipulations commonly used in studies of eye growth regulation, with consistent matches between gene expression patterns and visual manipulations, except for very short exposures. All three BMPs are up-regulated with imposed myopic defocus (positive lenses) and down-regulated with both imposed hyperopic defocus (negative lenses) and form-deprivation (imposed with diffusers). Second, the critical exposure duration required for accurate translation of these altered retinal experiences into biological signals at the level of the RPE is in the order of minutes to hours, depending on the nature of the visual manipulations.

As noted in the introduction, for human eyes, as well as a broad range of animal eyes, the optical (focusing) power of the eye and its length are not well matched at birth^2,3,14,15^. Nonetheless, most eyes actively grow towards emmetropia (perfect focus for distance objects) over the early postnatal period^2^. Eye growth appears to be regulated by both non-optical factors, such as genetically programmed organogenesis, and visual factors, as demonstrated experimentally, with manipulations such as applied in the current study^2–4^. There may also be other genetic influences within the signal cascade linking retinal activity to eye growth, as well as gene-environment interactions on many levels^3^. To-date, most progress towards understanding the mechanisms underlying eye growth regulation has been made on the output side, in studies targeting the choroid and sclera, with relatively little attention directed towards understanding the retinal signal pathway(s) and role of the RPE, and many unresolved questions remaining.

In this study, we used the posthatch chick as our model, in combination with two optical defocus paradigms and form-deprivation to study the roles of the RPE and BMPs. With defocusing lenses, the choroid may be induced to thicken or thin and overall eye growth, to slow or accelerate, according to the sign of the applied defocus (positive or negative resp.), until the applied defocus is neutralized, and homeostasis, restored. These close-loop conditions contrast with the open-loop condition imposed by form-deprivation, which induces choroidal thinning and accelerated eye growth, just as with negative lenses, but with no return to homeostasis. The roles for BMPs in eye organogenesis, morphogenesis and early development have already been documented in a variety of animal models, including chicks^16–21^. Previous observations of consistent BMP gene expression changes tied to altered eye growth e.g., up-regulation with slowed eye growth (positive lenses) and down-regulation with accelerated eye growth (negative lenses and form-deprivation), lead to our speculation that BMPs expressed by the RPE play key roles in a retina-sclera growth modulatory signal cascade. The results reported here lend further weight to this proposal.

There is on-going debate about whether the same or different mechanisms underlie the enhanced eye growth and myopia induced by negative lenses and form-deprivation^22^. For example, decreases in the rate of retinal dopamine release and metabolism are observed in both lens-induced and form-deprivation myopia, yet in pharmacological studies, the dopamine D2 antagonist, spiperone, and an M4 muscarinic antagonist, MT-3, have different effects on the responses to these two types of visual manipulations. The results from the current study argue that, at least at the level of the RPE, the same BMP signaling pathway is involved, although the retinal pathways involved in processing these altered retinal images may be different. We also cannot rule out divergence again downstream of the RPE, i.e., at the level of the choroid or sclera, although this seems less likely^12,22–24^.

The current study also offers insight into the roles of BMPs in the initiation of altered eye growth patterns. We were able to detect up-regulation, by over 200%, in BMP2 gene expression, as early as 15 minutes after the initiation of treatment with +10 and +20 D lenses. Taking into consideration the time for the retina to decode the applied defocus, as well as the time to activate the upstream BMP signaling pathway(s), including mRNA biosynthesis, 15 minutes seems a remarkably short time^2,25–28^. Nonetheless, given this response is tied to inhibited eye growth and that eyes appear to have only limited capacity to shrink, especially in the case of the chick, this rapid response has the functional advantage of preventing eyes from elongating excessively^29^. Down-regulation of BMP gene expression in response to −10 D lens and form-deprivation treatments was much slower to develop, first detectable after 2 h, although here also, as with positive lens treatments, gene expression changes precede detectable changes in eye size^9^. The latter timing tends to argue against these gene expression changes being secondary to the eye growth changes. These BMP gene expression changes were also enduring for all treatments, lasting up to 48 h, by which time changes in ocular dimensions are measurable, although in the case of the lens treatments used in the current study, not sufficient to eliminate the imposed defocus. Together, these findings suggest that the gene expression changes are a response to the imposed visual conditions rather than a product of induced altered growth patterns^9^.

In the context of myopia research, an ever-expanding list of biological molecules, in retina, choroid, and/or sclera, have been linked to eye growth regulation^2,6–8,30–35^. However, for many of them, detectable changes paralleled altered eye growth, as opposed to preceding them, leaving open the possibility that they were products of altered eye growth. Of these molecules, few also show consistent, bidirectional response patterns, as exhibited by BMP2, -4 and -7, in the form of RPE gene expression changes and of note, BMP gene expression changes are also closely tied to specific eye growth patterns. One of the few exceptions is ZENK, whose expression has been shown to increase in retinal glucagonergic amacrine cells with 2 h of positive lens treatment^34^. However, the effect of the negative lens treatment was not specific to this subset of amacrine cells and the temporal treatment parameter studied were more limited than in the current study. While the results of this ZENK expression study suggest that glucagonergic amacrine cells are one of the cellular components in the retinal signal pathway activated by myopic defocus, the identity of the signal molecule(s) acting on the RPE to effect observed BMP gene expression changes is as yet unresolved.

Interestingly, in the current study, all three types of treatments induced up-regulation of BMP gene expression with very short exposures. Similar transient gene and protein expression changes have also been reported in other related studies^34,36^. Could this response pattern represent the default response of this growth regulating signal pathway under ambiguous conditions? As noted earlier, in the presence of imposed defocus the visual and thus “retinal” experience will vary significantly over time under normal conditions (i.e., chicks free to move and look around). Thus to reliably decode the average magnitude and direction of retinal defocus, as input to the growth regulator, it makes sense that there be a minimum exposure and thus sampling time below fluctuations in defocus are ignored and a default response triggered. That this default response in BMP gene expression should be up- rather than down-regulation, as observed, and thus ocular elongation inhibited, is logical in serving to protect against early excessive eye growth from which recovery is generally limited^29^.

In summary, based on the results from the presented study in young chicks, we propose that the RPE and BMP genes play key roles in the ocular growth-modulation, representing components of a signal relay linking the retina with choroidal and scleral targets, which can be activated by both lens- and form-deprivation treatment. The critical exposure periods for visual manipulations, including imposed defocus, compatible with their accurate translation into biological signals in the RPE is in the order of minutes to hours, as reported here.

## Methods

### Animals and visual manipulations

White-Leghorn chickens were hatched from fertilized eggs sourced from University of California, Davis (Davis, CA), and raised under a 12 h light/12 h dark cycle in an Animal Facility at University of California, Berkeley (Berkeley, CA). At 14 days of age, chicks underwent monocular visual manipulations; specifically, they were fitted with either +10, +20, +30, or −10 D lenses, or diffusers, which were left in place for either 5, 15, 30, 60 minutes, 2 or 48 hours. The choice of lens powers precluded the possibility of rapid, complete and sustained compensation, via lenticular accommodation in the case of imposed hyperopic defocus (−10 D lens), and via choroidal thickening in the case of long exposures to imposed myopic defocus (+20 & +30 D lenses). Each treatment group comprises chicks from two or three independent repetitions of this experiment (3–4 per group in each experiment). Both the untreated contralateral (fellow) eyes of treated chicks and eyes of age-matched untreated chicks served as controls. Experiments were conducted according to the ARVO Statement for the Use of Animals in Ophthalmic and Vision Research and approved by the Animal Care and Use Committee (ACUC) at University of California, Berkeley, CA.

### Chick RPE tissue collection

RPE samples were collected as described previously^9,10^. Chicks were sacrificed at the end of experiments, eyes quickly enucleated and anterior ocular segments cut away to isolate posterior eye cups. The cups were immediately immersed in cold phosphate buffered saline (PBS) buffer, retinas then peeled off the RPE with forceps and the RPE collected by gently rinsing cells off the choroid with buffer. RPE cells were then spun down, lysed with RLT buffer from RNeasy Mini kits (Qiagen, Valencia, CA), and immediately stored at −80 °C for later use.

### RNA purification and real-time PCR

For all experimental manipulations, RPE gene expression changes were evaluated for three BMPs (BMP2, -4 and -7). Primer information and the calculation of gene expression levels of BMPs, and housekeeping gene GAPDH have been described in previous, related publications^9,10^. Total RNA was purified from RPE samples using RNeasy Mini kits (Qiagen), with on-column DNase digestion, according to the manufacturer’s protocol. RNA concentration and A_260_/A_280_ optical density ratio was then measured for quantification and quality control respectively with a spectrophotometer (NanoDrop 2000; NanoDrop Technologies, Inc., Wilmington, DE). Finally, RNA was reverse transcribed to cDNA (SuperScript III First-Strand Synthesis System for RT-PCR, Invitrogen, Carlsbad, CA). Quanti-Tect SYBR Green PCR Kits (Qiagen) were used for mRNA amplification, with a StepOnePlus Real-Time PCR System (Applied Biosystems). Melt curves were examined to verify the yield of single peak products. All real-time PCR reactions were performed in triplicate.

### Statistical analysis

Paired Student’s *t*-tests were used to compare gene expression data from lens-treated eyes with their fellows, as well as data from right and left eyes of the untreated group. Unpaired Student’s *t*-tests were used to compare differences in treatment-induced gene expression levels between groups fitted with diffusers versus −10 D lenses. Key results are also show graphically as bar graphs and heatmaps.

## Supplementary information

Supplemental Table 1

## References

[1] Flitcroft DI (2014). Emmetropisation and the aetiology of refractive errors. Eye (London, England).

[2] Wallman J, Winawer J (2004). Homeostasis of eye growth and the question of myopia. Neuron.

[3] Flitcroft DI (2013). Is myopia a failure of homeostasis?. Exp. Eye. Res..

[4] Hung GK, Ciuffreda KJ (2000). A unifying theory of refractive error development. Bull. Math. Biol..

[5] Stone RA, Khurana TS (2010). Gene profiling in experimental models of eye growth: clues to myopia pathogenesis. Vision Res..

[6] Rymer J, Wildsoet CF (2005). The role of the retinal pigment epithelium in eye growth regulation and myopia: a review. Vis. Neurosci..

[7] Nickla DL, Wallman J (2010). The multifunctional choroid. Prog. Retin. Eye Res..

[8] Rada JA, Shelton S, Norton TT (2006). The sclera and myopia. Exp. Eye Res..

[9] Zhang Y, Liu Y, Wildsoet CF (2012). Bidirectional, optical sign-dependent regulation of BMP2 gene expression in chick retinal pigment epithelium. Invest. Ophthal. Vis. Sci..

[10] Zhang Y, Liu Y, Ho C, Wildsoet CF (2013). Effects of imposed defocus of opposite sign on temporal gene expression patterns of BMP4 and BMP7 in chick RPE. Exp. Eye Res..

[11] Zhang Y, Wildsoet CF (2015). RPE and choroid mechanisms underlying ocular growth and myopia. Prog. Mol. Biol. Transl. Sci..

[12] McGlinn AM (2007). Form-deprivation myopia in chick induces limited changes in retinal gene expression. Invest. Ophthal. Vis. Sci..

[13] Troilo, D., Boisvert, N. & Nau, N. How is accommodation related to the development of refractive state? Evidence from experimental studies using animal models. *In:* Thorn, F., Troilo, D. & Gwiazda, J. *(eds), Myopia* 2000*: Proceedings of the VIII International Conference on Myopia Boston, MA: Myopia 2000, Inc; 254–258* (*2000*).

[14] Troilo D, Judge SJ (1993). Ocular development and visual deprivation myopia in the common marmoset (Callithrix jacchus). Vision Res..

[15] Wallman J, Adams JI, Trachtman JN (1981). The eyes of young chickens grow toward emmetropia. Invest. Ophthal. Vis. Sci..

[16] Huang J, Liu Y, Oltean A, Beebe DC (2015). Bmp4 from the optic vesicle specifies murine retina formation. Dev. Biol..

[17] Steinfeld J (2013). RPE specification in the chick is mediated by surface ectoderm-derived BMP and Wnt signalling. Development.

[18] Heermann S, Schutz L, Lemke S, Krieglstein K, Wittbrodt J (2015). Eye morphogenesis driven by epithelial flow into the optic cup facilitated by modulation of bone morphogenetic protein. Elife.

[19] Huang J, Liu Y, Filas B, Gunhaga L, Beebe DC (2015). Negative and positive auto-regulation of BMP expression in early eye development. Dev. Biol..

[20] Wordinger RJ, Clark AF (2007). Bone morphogenetic proteins and their receptors in the eye. Exp. Biol. Med. (Maywood).

[21] Belecky-Adams T, Adler R (2001). Developmental expression patterns of bone morphogenetic proteins, receptors, and binding proteins in the chick retina. J. Comp. Neurol..

[22] Morgan IG, Ashby RS, Nickla DL (2013). Form deprivation and lens-induced myopia: are they different?. Ophthalmic Physiol. Opt..

[23] Schaeffel F, Bartmann M, Hagel G, Zrenner E (1995). Studies on the role of the retinal dopamine/melatonin system in experimental refractive errors in chickens. Vision Res..

[24] Wildsoet C (2003). Neural pathways subserving negative lens-induced emmetropization in chicks–insights from selective lesions of the optic nerve and ciliary nerve. Curr. Eye Res..

[25] Schaeffel F, Wildsoet C (2013). Can the retina alone detect the sign of defocus?. Ophthalmic Physiol. Opt..

[26] Kaneda M (2013). Signal processing in the mammalian retina. J. Nippon Med. Sch..

[27] Burkhardt DA (2011). Contrast processing by ON and OFF bipolar cells. Vis. Neurosci..

[28] Masson G, Mestre D, Blin O (1993). Dopaminergic modulation of visual sensitivity in man. Fundam. Clin. Pharmacol..

[29] Zhu X, McBrien NA, Smith EL, Troilo D, Wallman J (2013). Eyes in various species can shorten to compensate for myopic defocus. Invest. Ophthal. Vis. Sci..

[30] Flitcroft DI (2012). The complex interactions of retinal, optical and environmental factors in myopia aetiology. Prog. Retin. Eye Res..

[31] Stone RA, Lin T, Laties AM, Iuvone PM (1989). Retinal dopamine and form-deprivation myopia. Proc. Natl. Acad. Sci. USA.

[32] McFadden SA, Howlett MH, Mertz JR (2004). Retinoic acid signals the direction of ocular elongation in the guinea pig eye. Vision Res..

[33] Seko Y, Shimokawa H, Tokoro T (1996). *In vivo* and *in vitro* association of retinoic acid with form-deprivation myopia in the chick. Exp. Eye Res..

[34] Fischer AJ, McGuire JJ, Schaeffel F, Stell WK (1992). Light- and focus-dependent expression of the transcription factor ZENK in the chick retina. Nat. Neurosci..

[35] Riddell N, Giummarra L, Hall NE, Crewther SG (2016). Bidirectional expression of metabolic, structural, and immune pathways in early myopia and hyperopia. Front. Neurosci..

[36] Schippert R, Brand C, Schaeffel F, Feldkaemper MP (2006). Changes in scleral MMP-2, TIMP-2 and TGFbeta-2 mRNA expression after imposed myopic and hyperopic defocus in chickens. Exp. Eye Res..

