# Supplementary material for: Retinal Defocus and Form-Deprivation Exposure Duration Affects RPE BMP Gene Expression
Source: Sci Rep. 2019 May 14;9:7332. doi: 10.1038/s41598-019-43574-z (PMC6517395; doi:10.1038/s41598-019-43574-z)
Supplement: Supplementary file 1 — Supplemental Table 1 [file 41598_2019_43574_MOESM1_ESM.docx]

**Retinal Defocus and Form-Deprivation Exposure Duration Affects RPE BMP Gene Expression**

Yan Zhang, Eileen Phan, Christine F. Wildsoet

School of Optometry, University of California, Berkeley, Berkeley, CA

**Corresponding author**:

Yan Zhang, M.D., Ph.D.

E-mail address: yanzhang@berkeley.edu

Mailing address: 592 Minor Hall, School of Optometry, University of California, Berkeley,

Berkeley, CA 94720

Supplemental Table 1. +10, +20, +30, -10 D lens and form-deprivation-induced BMP gene expression changes expressed as mean ratios of levels in treated eye/contralateral eye (%, SEMs in brackets).

|  |  | 5 min  (%, n = 11) | 15 min  (%, n = 11) | 30 min  (%, n = 10) | 60 min  (%, n = 11) | 2 h  (%, n = 9) | 48 h  (%, n = 8) |
| --- | --- | --- | --- | --- | --- | --- | --- |
|  | BMP2 | 129.8 [29.2] | 295.4 [58.5]^***^ | 382.0 [93.0]^**^ | 433.5 [61.5]^*^ | 635.3 [184.9]^**^ | 738.7 [121.3]^***^ |
| +10 D | BMP4 | 187.2 [88.4] | 188.0 [27.5]^**^ | 225.0 [68.7]^*^ | 247.6 [42.6]^*^ | 286.7 [57.3]^***^ | 357.9 [58.6]^***^ |
|  | BMP7 | 157.5 [73.2] | 128.2 [16.3] | 135.7 [38.7] | 125.7 [18.5] | 175.7 [39.6]^*^ | 140.2 [13.0]^*^ |
|  |  | 5 min  (%, n = 10) | 15 min  (%, n = 9) | 30 min  (%, n = 11) | 60 min  (%, n = 9) | 2 h  (%, n = 12) | 48 h  (%, n = 11) |
|  | BMP2 | 121.4 [33.5] | 241.5 [43.3]^**^ | 303.1 [85.4]^*^ | 394.9 [157.8]^*^ | 599.6 [254.3]^**^ | 703.3 [150.6]^***^ |
| +20 D | BMP4 | 115.1 [18.9] | 188.1 [28.1]^**^ | 269.4 [99.1] | 209.4 [61.7] | 253.3 [61.3]^*^ | 402.2 [73.3]^***^ |
|  | BMP7 | 81.4 [9.6] | 139.0 [18.1] | 191.2 [63.2] | 140.2 [36.9] | 106.0 [16.8] | 129.4 [16.8] |
|  |  | 5 min  (%, n = 10) | 15 min  (%, n = 10) | 30 min  (%, n = 11) | 60 min  (%, n = 10) | 2 h  (%, n = 12) | 48 h  (%, n = 11) |
|  | BMP2 | 291.4 [52.9] ^**^ | 108.9 [21.0] | 155.8 [26.7] | 244.7 [73.5] | 318.1 [126.9] | 582.4 [102.5]^***^ |
| +30 D | BMP4 | 241.7 [42.7] ^***^ | 103.6 [14.1] | 144.5 [23.6] | 154.4 [39.7] | 175.1 [45.3] | 332.5 [35.4]^***^ |
|  | BMP7 | 162.8 [23.2]^**^ | 89.6 [11.4] | 113.9 [18.5] | 140.4 [39.6] | 120.5 [22.8] | 138.2 [15.6] |
|  |  | 5 min  (%, n = 10) | 15 min  (%, n = 14) | 30 min  (%, n = 11) | 60 min  (%, n = 9) | 2 h  (%, n = 9) | 48 h  (%, n = 8) |
|  | BMP2 | 181.3 [32.2] | 206.4 [37.8] | 141.5 [27.0] | 96.7 [23.5] | 30.6 [8.4]^**^ | 22.9 [4.7]^**^ |
| -10 D | BMP4 | 142.3 [23.7] | 172.7 [36.6] | 142.8 [24.7] | 107.4 [30.0] | 45.2 [9.9]^*^ | 48.5 [10.7]^*^ |
|  | BMP7 | 109.3 [12.3] | 146.0 [27.4] | 129.9 [16.8] | 117.0 [25.3] | 70.8 [11.2]^*^ | 74.3 [9.5]^*^ |
|  |  |  | 15 min  (%, n = 11) | 30 min  (%, n = 12) | 60 min  (%, n = 12) | 2 h  (%, n = 9) | 48 h  (%, n = 8) |
|  | BMP2 | 158.0 [20.6] | 213.8 [46.1] | 231.5 [114.1] | 94.2 [15.7] | 27.4 [5.6]^**^ | 11.6 [2.9]^***^ |
| FD | BMP4 | 125.9 [15.7] | 184.7 [38.9] | 225.1 [106.2] | 90.4 [12.3] | 67.6 [12.6]^*^ | 31.5 [5.9]^***^ |
|  | BMP7 | 89.2 [11.5] | 141.0 [23.5] | 216.3 [97.1] | 120.7 [13.8] | 103.4 [11.7] | 55.6 [8.6]^**^ |

FD, form-deprivation; * *p* < 0.05, ** *p* < 0.01, *** *p* < 0.001.
